# Crystal Structure of Enhanced Green Fluorescent Protein to 1.35 Å Resolution Reveals Alternative Conformations for Glu222

**DOI:** 10.1371/journal.pone.0047132

**Published:** 2012-10-16

**Authors:** James A. J. Arpino, Pierre J. Rizkallah, D. Dafydd Jones

**Affiliations:** 1 School of Biosciences, Cardiff University, Cardiff, United Kingdom; 2 School of Medicine, Cardiff University, Cardiff, United Kingdom; Berlin Institute of Technology, Germany

## Abstract

Enhanced Green Fluorescent Protein (EGFP) is one of the most widely used engineered variants of the original wild-type Green Fluorescent Protein. Here, we report the high resolution (1.35 Å) structure of EGFP crystallised in its untagged sequence form that reveals the combined impact of the F64L and S65T, that give rise to improved folding and spectral characteristics. The overall structure of EGFP is very similar to wt GFP, forming the classical β-barrel fold with the chromophore containing helix running through the core of the structure. Replacement of Phe64 with Leu in EGFP results in subtle rearrangement of hydrophobic core packing close to the chromophore including the reduction in surface exposure of two hydrophobic residues. Replacement of Ser65 with Thr has a significant impact on the local hydrogen bond network in the vicinity of the chromophore. Detailed analysis of electron density reveals that several residues close to the chromophore occupy at least two distinct conformations. This includes Glu222 that defines the charged state on the chromophore, with the two conformations having slightly different effects on the hydrogen bond network surrounding the chromophore. Hence, the reported high-resolution structure of EGFP has provided a long overdue molecular description of one of the most important fluorescent protein variants currently in general use.

## Introduction

Since it's discovery [1] and subsequent use as a genetic protein tag [2], Green Fluorescent Protein (GFP) from *Aequorea victoria* has become one of the most important and powerful tools in cell biology [3], [4]. Its intrinsic fluorescence without the requirement of any additional co-factors or substrates allowed for the first time genetically encoded defined tagging and monitoring of any target protein in the cell. GFP and other since discovered fluorescent proteins from different organisms [5] are fluorescent due to covalent rearrangement of contiguous amino acids [3]. In the case of wt GFP Ser65, Tyr66 and Gly67 main-chain atoms cyclise after the protein folds to form, in the presence of O_2_, the highly conjugated planar *p*-hydroxybenzylideneimidazolinone chromophore [6]. The crystal structure of wild-type GFP (wt GFP) [7], [8] revealed that the chromophore is packed within the core of the GFP β-barrel structure protecting it from quenching through water dipoles, paramagnetic oxygen or *cis-trans* isomerisation. The spectral properties of the GFP chromophore are further tuned through non-covalent interactions with neighboring residues [3].

The original wt GFP had several major drawbacks that reduced its effectiveness as a tool for cell imaging [3]. Its folding efficiency and thus fluorescent signal drops dramatically at physiologically relevant temperatures such as 37°C, its maturation rate is very slow and it had a strong tendency to aggregate. Two separate excitation peaks were also observed due to the coexistence of neutral (λ_ex_∼395 nm) and phenolate (λ_ex_∼490 nm) forms of the chromophore [9]. Excitation at ∼490 nm was preferred as its lower energy is less damaging to the cell. However, the 490 nm excitation wavelength was only a minor contributor to fluorescence (∼15% compared to excitation at ∼395 nm). Protein engineering has solved many of the problems associated with wt GFP so facilitating its rapid and wide spread use [3]–[5], [10]. One of the first and most important engineered versions of wt GFP was enhanced GFP (EGFP) [11], [12]. EGFP has greater folding efficiency (increased fluorescence due to a higher proportion of correctly folded protein) at 37°C, has a single excitation peak at ∼490 nm and has been codon optimized for expression in mammalian hosts. Two mutations that generate EGFP, F64L and S65T, contribute to these improved properties. S65T is considered essential for suppressing the 395 nm excitation peak through modulation of the ionized state of nearby Glu222, whilst the F64L mutation is responsible for improved folding efficiency at 37°C. In wt GFP, Ser65 donates a hydrogen bond to the carboxyl group of Glu222 and promotes the deprotonated form. Electrostatic repulsion prevents both the Glu222 and the chromophore occupying the same anionic state.

EGFP is still one of the most widely utilized of all the GFP variants but its native sequence structure in the absence of any tag has not been determined to high resolution so as to fully understand the impact and context of the F64L and S65T mutations. The only available structure of EGFP-like protein available in the PDB was determined only very recently at the slightly lower resolution of 1.5 Å, and with an N-terminal protein purification affinity [His] tag present [13], accession code 2Y0G. Here we report the crystal structure of the native EGFP without any purification tags present to 1.35 Å resolution. Analysis of the structure suggests that the mutations have subtle yet important effects on the side chain packing and hydrogen bond network surrounding the chromophore. The high resolution data has identified several residues close to the chromophore that exist in multiple conformations. These include Glu222, which defines the charge character of the chromophore, Leu18 and Leu44. Our work independently confirms the presence of alternate conformations previously observed for Glu222 but with significant differences in side chain placement in the additional conformer. The observation of alternate conformers for Leu18 and Leu44 has not been previously reported to our knowledge.

## Results and Discussion

### General characterisation of EGFP

EGFP was purified in its native sequence form, encompassing residues 1-Met-Val-Ser to Leu-Tyr-Lys-238 without any affinity purification tag attached at the N- or C-termini. The spectral characteristics of the pure protein were similar to those already reported for EGFP [14], with λ_ex_ and λ_em_ of 488 nm and 511 nm, respectively, ε of 55 mM^−1^ cm^−1^, a quantum efficiency of 0.6 and brightness of 33 mM^−1^ cm^−1^. Size exclusion chromatography (Methods S1) confirmed EGFP was predominantly monomeric (Figure S1).

### Crystal structure of EGFP

The crystal structure of EGFP showing residues Lys3 to Leu231 was determined as described in the Methods and Materials. Crystals grew in the space group P2_1_2_1_2_1_ and contained a single molecule in the asymmetric unit. The structure was determined to a resolution of 1.35 Å and refined to an R and R_free_ of 12.8% and 16.8%, respectively (Table 1). The final refinement statistics and model geometry fall within the expected range (Table 1).

**Table 1:** Cystallographic statistics.

Data reduction statistics	
Beamline	Diamond I02
Wavelength (Å)	*0.97988*
Space group	*P*2(1)2(1)2(1)
a (Å)	51.1
b (Å)	62.2
c (Å)	69.6
Resolution range (Å)	46.42 - 1.35
Total reflections measured	225405
Unique reflections	49477
Completeness (%) (last shell)	99.8 (99.5)
I/σ (last shell)	5.0 (2.0)
R(merge)^a^ (%) (last shell)	9.3 (38.6)
B(iso) from Wilson (Å^2^)	10.6

Refinement statistics	
Protein atoms excluding H	2385
Solvent molecules	393
R-factor^b^ (%)	12.8
R-free^c^ (%)	16.8
Rmsd bond lengths (Å)	0.017

Ramachandran plot statistics	
Rmsd angles (°)	2.0
Core region (%)	92.7
Allowed region (%)	7.3
Additionally allowed region (%)	0.0
Disallowed region (%)	0.0

a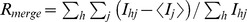.

b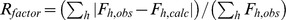.

c *R_free_* is calculated from a set of 5% randomly selected reflections that were excluded from refinement.

**Figure 1:**
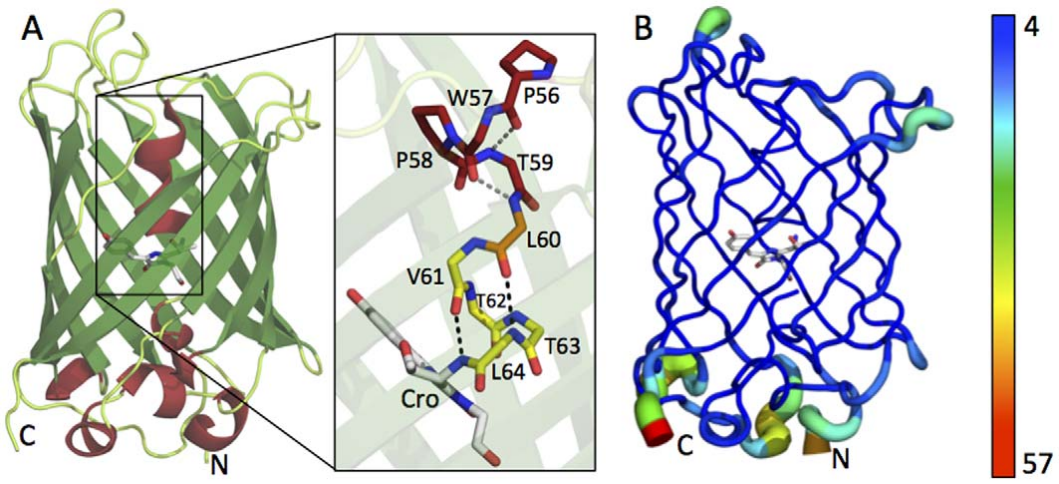
Structure of EGFP. (A) Ribbon representation of EGFP highlighting β-strands in green, helices in red and loops in pale green. **Inset**, Stick representation of the central helix forming residues (main chain only) in the 3_10_ conformation (dark red) and in the α conformation (yellow). Residue Leu60 (orange) is involved in hydrogen bonding in the 3_10_ portion of the helix (grey dashed lines) and the α portion of the helix (black dashed lines). (B) B-Factor putty representation of EGFP with chromophore shown as sticks. The B-factor color scale is shown to the right. The regions with high B-factors (shown as bulges) are: K3 to G10 (N-terminus); G115 to D116; K131 to G134; A154 to I161; P187 to P196; K209 to E213; G228 to L231 (C-terminus).

The crystal structure of EGFP displays the traditional β-barrel structure with the chromophore located in the core of the protein (Figure 1A). Secondary structure assignment using DSSP revealed that 11 strands make up the β-barrel core, corresponding to 47% of protein secondary structure. Only 13% is helical, with the core helix containing the chromophore being a mixture of the 3_10_ and α conformations. Whilst secondary structure assignment using DSSP identified the 3_10_ helix in the core of the protein (Pro56 – Leu60) it failed to identify residues Val61 – Leu64 as being α-helical in nature. Structural analysis of the residues in the central helix identify an i to i+3 hydrogen bonding pattern between residues Pro56 to Leu60 corresponding to a 3_10_ helix, despite proline being considered as a typical helix breaker, whilst an i to i+4 hydrogen bonding pattern is seen from residues Leu60 to Leu64 corresponding to an α-helix (Figure 1A). The remaining 40% of the structure is comprised of coil mostly from loops located at the two ends of the β-barrel structure (Figure 1A). A full list of secondary structure assignments can be found in Table S1.

A B-factor ‘putty’ (or ‘sausage’) representation of EGFP (Figure 1B) highlights increased B-factor values for residues at the N- and C-termini and in several loops connecting ordered secondary structures. This may be an indication of increased mobility in these regions and could be considered as possible target sites for future protein engineering endeavors. Overall the B-factor values observed for the higher resolution EGFP, 4EUL, are slightly lower in comparison to those observed for the previously determined lower resolution structure, 2Y0G, providing increased confidence in the placement of side chains in the structure determined here. This has implications in terms of the certainty of alternate side chain conformations observed for EGFP (*vide infra*).

**Figure 2:**
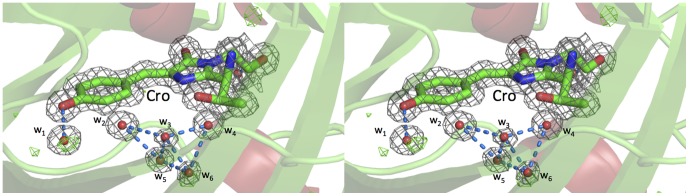
Chromophore local environment. Stereo view of the chromophore local environment with 2F_o_-F_c_ electron density map contoured to 1σ, highlighting the presence of several conserved water molecules and hydrogen bonding interactions (blue dashed lines). Whilst waters W_1_ to W_4_ are present in both wt GFP and S65T GFP W_5_ and W_6_ are absent. W_1_ to W_6_ are all present in the lower resolution EGFP structure 2Y0G. W_1_ to W_6_ refer to solvent molecules 413, 442, 564, 421, 494 and 547 respectively in structure 4EUL.

Despite the mature chromophore requiring protection from the external environment to maintain fluorescence several structured water molecules are also found within the core of the protein (Figure 2), some of which are critical to the fluorescent properties. Whilst all of these waters superimpose with waters from the lower resolution EGFP structure 2Y0G, two of the waters (W_5_ and W_6_) are absent in both structures for wt GFP (1GFL) and for S65T GFP (1EMA).

### Comparison of EGFP structures with wild-type GFP and S65T GFP structures

**Figure 3:**
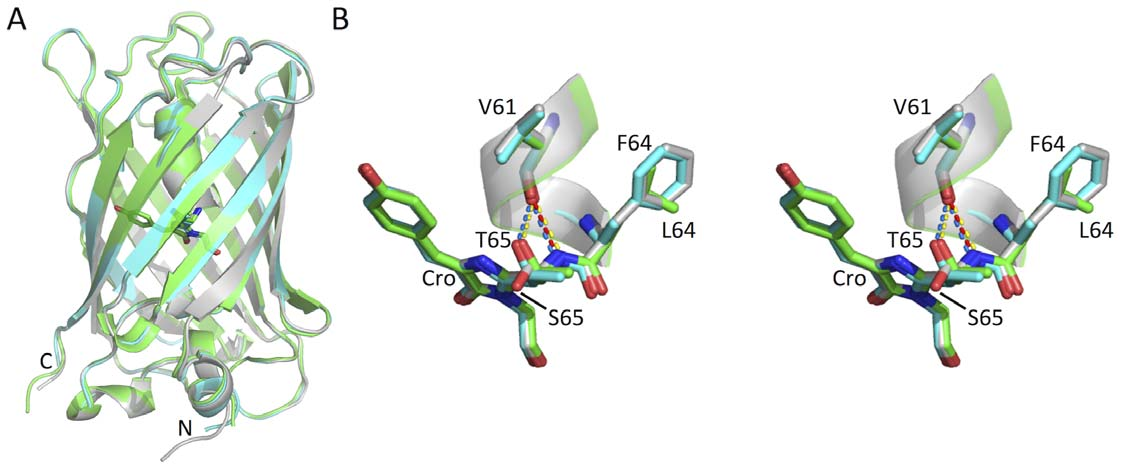
Structural comparison of wt GFP, S65T GFP and EGFP. (A) overlay of EGFP (green), wt GFP (grey) and S65T GFP (cyan). (B) Stereo view of the influence of the S65T mutation on local hydrogen bond network. EGFP, wt GFP and S65T GFP coloured as in A. Hydrogen bonds associated with EGFP, wt GFP and S65T GFP are yellow, red and blue dashed lines respectively.

Superpositioning of the structure obtained for EGFP with that of wt GFP (PDB entry 1GFL [7]) and a S65T GFP mutant (PDB entry 1EMA [8]) shows that the overall structures are very similar (Figure 3A); the RMSD over the backbone and all atoms of EGFP and wt GFP is 0.40 Å and 1.03 Å respectively, whilst the RMSD over backbone and all atoms of EGFP and S65T GFP are 0.29 Å and 0.85 Å, respectively. This indicates that the F64L and S65T mutations do not have a significant effect on the overall protein structure but have more subtle effects. Secondary structure analysis revealed the boundaries between the different elements in wt GFP and EGFP (Table S1) are very similar.

Superposition of the present model, 4EUL, with the lower resolution 2Y0G structure with an extended N-terminal His-tag (MAHHHHHHGHHH) sequence, reveals a RMSD of 0.59 Å when all common atoms are considered, including side chains. This indicates that both structures are largely identical. Thus, the presence of the longer than normal His tag sequence element in 2Y0G does not appear to greatly influence the overall structure. Both suffer from disordered termini. Whilst there are a number of identical residues in both versions of the EGFP structure that have been refined with multiple conformers, there are several that are only present in one or the other structure. These will be discussed in more detail below.

### Influence of F64L on EGFP structure

**Figure 4:**
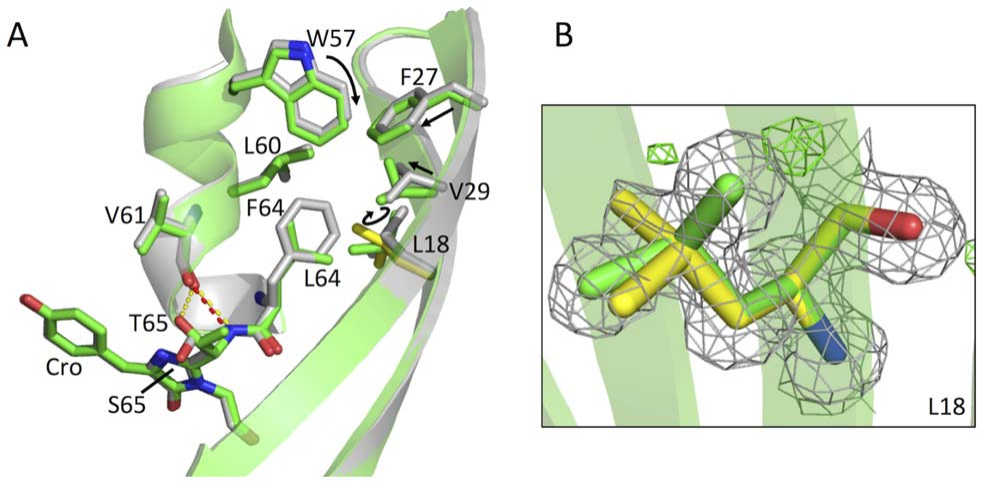
Effect of F64L mutation on EGFP structure. (A) Structural effects of the F64L mutation on the positions of L18, F27, V29, W57 and L64. EGFP and wt GFP coloured as in A. (B) 2F_o_-F_c_ electron density map, contoured to 1σ, of the L18 double conformation. Each conformer is coloured green (fit to 70% occupancy) and yellow (fit to 30% occupancy).

Given the high resolution of the EGFP structure, the exact placement of side chains can be defined with high confidence. The F64L mutation confers increased folding efficiency to GFP at 37°C but the structural consequences of this mutation in the context of the S65T mutation have not been fully investigated. The most obvious effect of the F64L mutation comprises the exchange of the bulky and buried phenylalanine side chain for a smaller leucine side chain in the central chromophore containing helical structure (Figure 4A). The substitution causes β-strand 2 to pack tighter with the core. The largest observed variation between wt GFP and EGFP was centred on residue Val29, with deviation of 1.37 Å across all atoms (Figure 4A). Val29 shifts closer to the chromophore upon the additional space being made available by loss of the aromatic Phe64 side chain. Residue Leu18 also shifts positions with the electron density only fully satisfied by modelling two alternate side chain conformations (Figure 4B), both of which differ from the side chain conformation of wt GFP Leu18 (Figure 4A). To satisfy the electron density the two conformations were modelled with an occupancy of 0.7 for conformer A and 0.3 for conformer B (Figure 4). Conformer A and B of Leu18 have observed RMSDs of 0.53 Å and 1.22 Å between wt GFP and EGFP across all atoms respectively. Both conformers exhibit a rotation of the Leu18 isobutyl side chain away from the edge of the β-barrel towards the core of the protein (Figure 4A). Whilst the lower resolution 2Y0G structure also shows rotation of the Leu18 isobutyl side chain away from the edge of the β-barrel it was not modelled by two conformers as seen in the present structure (Figure S5).

There is also a slight shift of residue Trp57 away from the surface of the protein towards Leu64, resulting in an RMSD over the side chain atoms of 0.39 Å (Figure 4A), decreasing its solvent accessible surface area in EGFP (12.8 Å^2^) with respect to wt GFP (15.2 Å^2^). Trp57 lies in the proline rich PVPWP pentapeptide sequence found in a variety of different proteins and, along with Val55 has been reported to be essential for function as mutation to other residues render EGFP non-fluorescent [15], [16]. The bulky side chain of Phe27 also moves towards the core of the protein with an RMSD over the side chain atoms of 0.41 Å resulting in a decreased solvent accessible surface area in EGFP (1.7 Å^2^) with respect to wt GFP (2.6 Å^2^) (Figure 4A). The repositioning of these residues could potentially influence the folding of EGFP at 37°C through better packing of the hydrophobic residues surrounding the central helix and of the central helix itself, and by reducing surface exposure of hydrophobic residues.

### Influence of S65T on EGFP structure

The S65T mutation has proved to be a more general mutation that can be transplanted to other green fluorescent protein variants to alter their spectral properties through removal of the 395 nm and promotion of the ∼490 nm excitation λ_ex_ [17], [18], increase the rate of oxidation during chromophore maturation and increase the brightness of the fluorescent proteins [17]. Analysis of the local environment around the chromophore can explain the molecular basis for the observed spectral properties. Replacement of Ser65 with Thr results in the hydroxyl group of Thr65 in EGFP occupying a different position from the corresponding hydroxyl group of Ser65 in wt GFP (Figure 3), probably as a result of steric effects due to the additional methyl group of Thr65. The Thr65 side chain in the EGFP structure determined here is in the same orientation as in the previously determined S65T-GFP structures [18] (Figure 3B).

**Figure 5:**
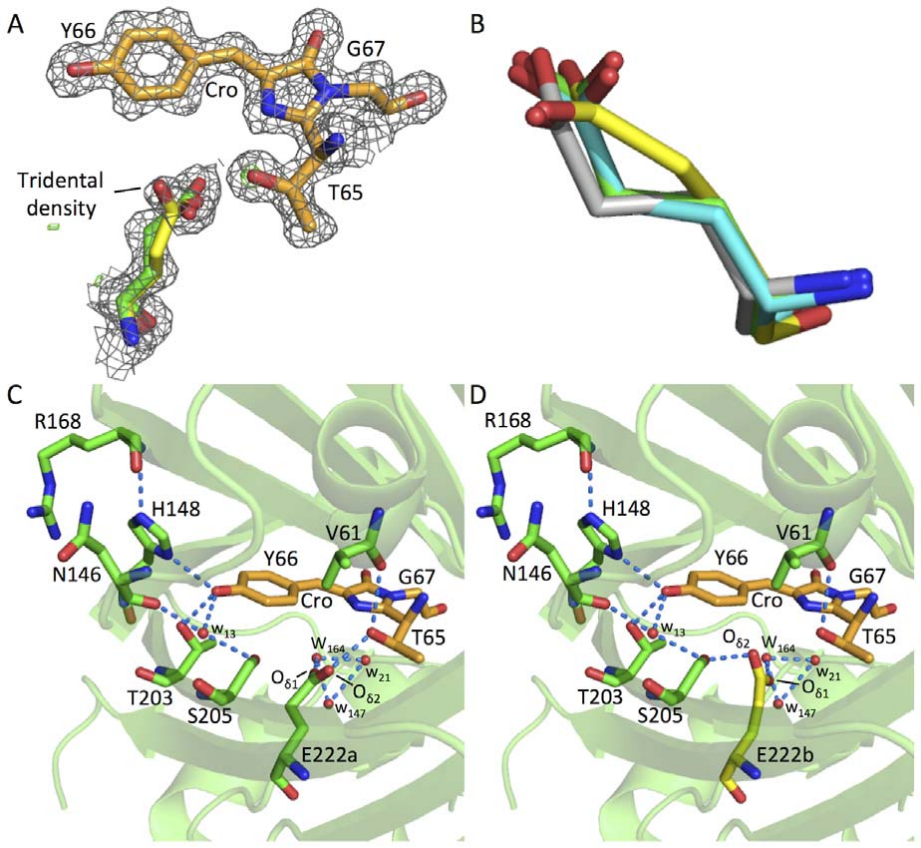
Effect of S65T mutation on Glu222 conformation and hydrogen bonding network. (A) 2F_o_-F_c_ electron density map for the chromophore and E222 (sticks) contoured to 1σ. Tridentate density for E222 modelled to two side chain conformations fit to 70% (E222a) and 30% (E222b) occupancy. (B) Superposition of E222 sidechain conformer A (E222a; green) and B (E222b; yellow) with the E222 side chain from wt GFP (1GFL; grey) and S65T GFP (1EMA; cyan). (C & D) The hydrogen bond network for each E222 conformer is indicated as dashed blue lines. The O_δ1_ alternatively donates a hydrogen bond to T65 OH (E222a; C) or S205 OH (E222b; D).

In contrast to other S65T-GFP structures the electron density of the Glu222 carboxylate side chain in EGFP suggests that it occupies two distinct conformations (Figure 5A). The electron density difference map produced after molecular replacement and structural refinement displays a tridentate density for Glu222 (Figure 5A), which was successfully modeled as two conformations of the carboxylate, with substantial difference in the placement of atoms along the side-chain (Figure 5B). The rationale behind the Glu222 double conformer in EGFP is described in Figure S2. The occupancy of conformers A and B were set to 70% and 30%, respectively, in order to satisfy the electron density for this residue. The Glu222 conformer A matches very closely to the position of Glu222 seen in the S65T GFP mutant but differs significantly from that observed for wt GFP (Figure 5B). This highlights the role of residue 65 in defining the positioning of Glu222. However, conformer B represents a new and different side-chain positioning for this residue, leaving the carboxylate group orientated in a similar direction (but not superimposable) with the Glu222 carboxylate group from wt GFP (Figure 5B). Tridentate density for Glu222 has only been observed to our knowledge once before in a GFP-derived variant, the recently determined His-tagged EGFP structure 2Y0G [13]. While conformer A in both these independently determined structures are very similar, conformer B shows obvious differences. With regards to Y20G, the tridentate electron density was refined to show changes largely related to the carboxyl group and not the side-chain as a whole (Figure S3), as observed here for 4EUL (Figure 5B and Figure S3). The hydroxyl group of Thr65 now donates a hydrogen bond to the backbone carbonyl of V61 instead of Glu222 (Figure 3B). However, the hydrogen bonding network related to the carboxylate of Glu222 depends on the conformation sampled, as outlined in Figure 5. Thus, we have provided important independent proof that Glu222 can exist in two alternate conformations, albeit with slight discrepancies concerning the alternate B conformation, and with the higher resolution structure can position the atoms of the alternate conformer of Glu222 with higher confidence.

The reason for promotion of the anionic chromophore over the neutral form is thought to be a result of disruption of hydrogen bonding with charged Glu222 on mutation of Ser65 to Thr [3], [18], [19]. This in turn prevents ionization within the core of the Glu222 carboxylate group so removing an electrostatic clash with the anionic form of the ground state chromophore. In both conformations Glu222 O_δ1_ is hydrogen bonded to two conserved water molecules. With respect to Glu222 O_δ2_, in one conformation it donates a hydrogen bond to the hydroxyl group of Thr65 (Figure 5C) and in the alternate conformation it donates a hydrogen bond to the hydroxyl group of Ser205 (Figure 5D). In order for Glu222 to donate hydrogen bonds its carboxylate group must be protonated and therefore neutral. This allows charge stabilization on the deprotonated phenol group of the chromophore by hydrogen-bonding interactions from His148, Thr203 and a conserved water molecule coordinated between the backbone carbonyl group of Asn146 and the side chain hydroxyl group of Ser205 (Figure 5C & 5D). The neutral charge on Glu222 also removes any potential electrostatic clashes between the negative charges on Glu222 and the chromophore, thus allowing the chromophore to be deprotonated in the ground state. This explains why EGFP has a single excitation peak corresponding to the deprotonated state.

**Figure 6:**
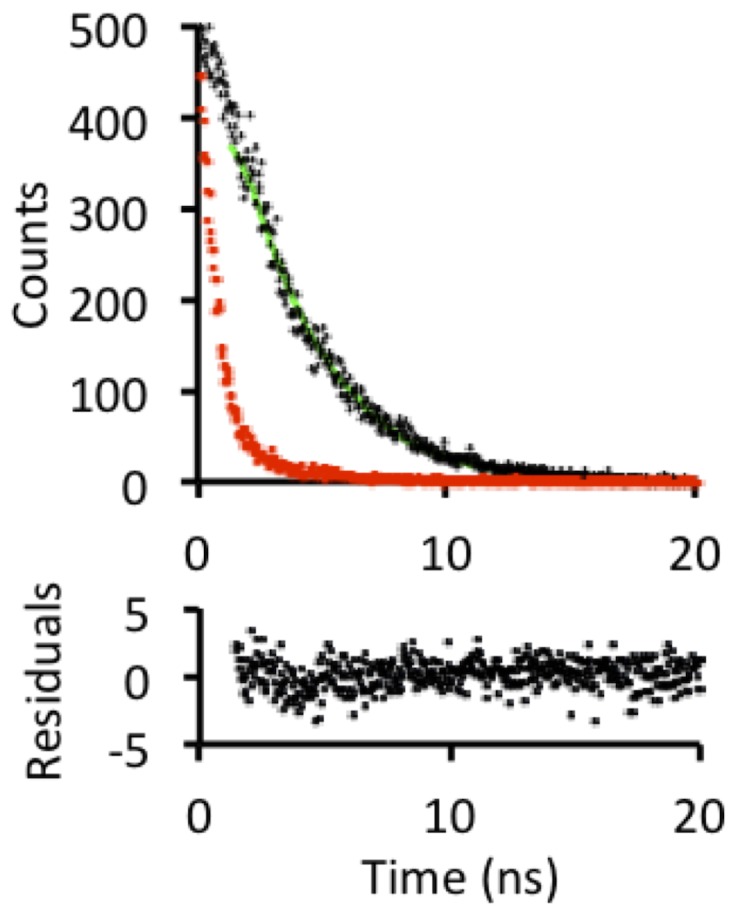
Fluorescence lifetime analysis of EGFP. Photon counts (black dots) measured over 20 ns were fit to a single exponential decay function (green line) with the instrument response function shown as red dots. Residuals for the single exponential decay fit to the data are plotted below the decay curve.

However, given the heterogeneity in the local environment of the chromophore due to the alternate conformations of Glu222 it would be expected that this may be reflected by spectral heterogeneity. This is not the case as is evident from single exponential fluorescence lifetime decays (Figure 6); the measured fluorescence lifetime was 2.54 ns, similar to that reported previously for EGFP [20]. Therefore, small alterations in Glu222 conformation are unlikely to have profound effects on the electrostatic environment surrounding the chromophore and thus the fluorescence properties. The two side chain conformations observed for Glu222 could be a crystallographic artifact and both may not be populated in solution. This is unlikely given that the residue is buried within the interior of the protein and alternate conformations for this residue have been observed in a lower resolution structure of the His-tagged EGFP protein [13]. Alternatively both conformations may exist but could be in a rapid dynamic equilibrium and transiently exchanging between the two conformations. A third possibility could be that upon folding the Glu222 side chain is trapped in one conformation or the other. Further structural analysis by NMR could potentially identify and measure Glu222 side-chain exchange rates in solution to confirm the two conformations observed in the crystal structure.

### Other residues exhibiting multiple conformers

**Figure 7:**
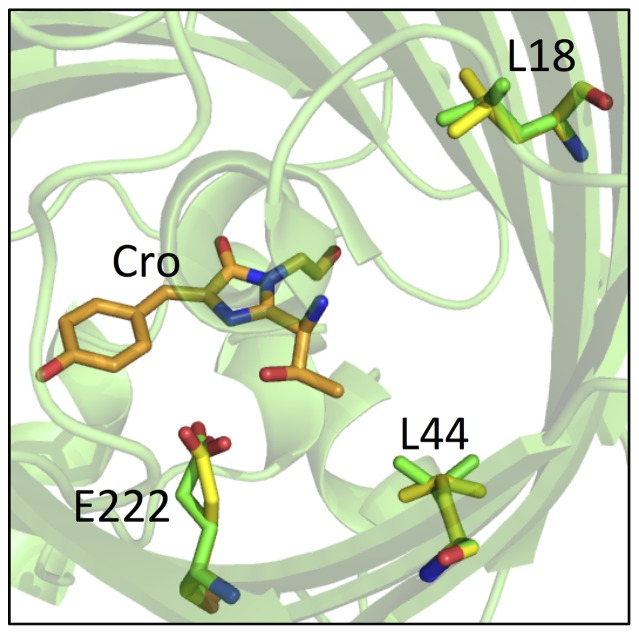
Buried residues with multiple conformers close to the chromophore. Cartoon representation of EGFP (green) showing the chromophore (Cro; orange sticks) in close proximity to residues L18, L44 and E222 all of which have been modeled to two conformers; conformer A (green sticks) or conformer B (yellow sticks) were modeled to an occupancy of 70% or 30% respectively.

In addition to Leu18 and Glu222 that lie close in space to the chromophore (Figure 7), electron density maps indicated other residues occupied multiple conformations including Ser30, Thr43, Leu44, Gln80, Thr97, Lys101, Asp102, Lys113, Asp117, Glu124, Met153, Glu172, Tyr182, Gln184 and Thr186 (Figure S4). Whilst the majority of these residues are surface exposed and potentially have the ability to sample multiple conformations freely, residues Leu18 and Leu44 are buried in the core of the protein in close proximity to Glu222 and the chromophore (Figure 7). Both Leu18 and Leu44 are modeled by single conformations in the lower resolution 2Y0G (Figure S5). The electron density for all three residues was best fitted when one conformer occupancy was set to 70% and the other conformer occupancy set to 30%. The implications of the dual conformers in terms of fluorescence characteristics, including whether one represents a fluorescent and the other a non-fluorescent state, are not currently known.

Comparison of 4EUL with the lower resolution 2Y0G structure identified a number of common double conformers observed between the two structures (S30, T43, K101, D102, D117, E124, E172, Q184, T186 and E222). Additional solvent exposed residues were modeled as double conformers in 2Y0G that were not observed in 4EUL (I47, K79, K107, N164, D190 and D197). Likewise a number of double conformers were modeled in 4EUL that were not observed in 2Y0G (L18, L44, Q80, T97, K113, M153). Leu18, Leu44 and Glu222 aside, given the solvent exposed nature of these residues and the difference in crystallisation conditions (4EUL: 0.1M MES/NaOH, pH 6.5, 200 mM calcium acetate, 20% (w/v) PEG8000. 2Y0G: 50 mM HEPES, pH 8.2, 24% (w/v), 50 mM magnesium chloride, PEG4000, 10 mM β-mercaptoethanol) the difference in double conformers modeled between the two structures are potentially crystallographic artifacts arising due to fixing of particular conformation populations in the crystal during random rotations around the side chain σ-dihedral bonds.

In conclusion, we have determined the structure of the native sequence to 1.35 Å resolution of the widely used and commercially important enhanced Green Fluorescent Protein. While the core structure and fold of EGFP is very similar to the wild-type GFP, the introduction of the F64L and S65T have subtle yet important effects on the properties of EGFP. These include altered core packing arrangements close to the chromophore and altering hydrogen bonding and charged states of residues close to the chromophore. These changes in turn give rise to the important properties of EGFP that make such a useful tool compared to the wt GFP: better fluorescence excitation characteristics and improved folding at 37°C. Comparison of the EGFP structure determined here (4EUL) with that of an extended N-terminal His-tagged version recently determined at slightly lower resolution (2Y0G) shows that the structures are very similar so the artificial affinity tag is having little overall effect on structure. However, the present higher resolution structure with slightly lower overall B-factor values increases confidence of side chain placement during the model refinement, which becomes crucial when alternate conformations are observed. Our work independently confirms the recent observation of an alternate conformation of Glu222 but here the side chain placement of the alternate conformer is different to that in the lower resolution structure. In addition, alternate conformations were observed for two residues close to the chromophore that were absent from the lower resolution structure suggesting that the presence of alternate conformations go beyond that of just Glu222. Thus, the observed multiple conformations for residues close to the chromophore, including the chromophore charge defining residue Glu222 is intriguing and may be having a yet unknown impact on the fluorescent properties of EGFP.

## Materials and Methods

### Protein production and purification

The production and subsequent purification of EGFP was performed as follows. LB Broth (15 ml) supplemented with 100 µg/ml ampicillin was inoculated with a single colony of *Escherichia coli* BL21 (DE3) Gold containing the plasmid pNOM-XP3-*egfp* to generate a starter culture and incubated overnight at 37°C. A 1/200 dilution of the starter culture was used to inoculate 1 l LB broth supplemented with 100 µg/ml ampicillin and grown at 37°C until an optical density of A_600_ = 0.4 was achieved. Protein expression was induced by the addition of 1 mM IPTG. The culture was incubated for 24 hrs at 37°C. The 1 l culture was then harvested by centrifugation (3000× g for 20 mins) and the pellet resuspended in 20 ml 50 mM Tris-HCl pH 8.0 (Buffer A) supplemented with 1 mM PMSF and 1 mM EDTA. The cells were lysed by French press using a chilled pressure cell. The lysate was then centrifuged (20000 rpm in Beckman JA20 rotor for 30 mins) to pellet any cell debris and the supernatant was decanted and stored at 4°C. The cell lysate was applied to a Q-Sepharose (GE healthcare) ion exchange column and elution monitored at 280 nm and 488 nm. Pooled fractions were then subjected to ammonium sulphate precipitation to further purify and concentrate the protein sample. An initial ammonium sulphate concentration of 45% (w/v) was used to precipitate unwanted proteins from solution. Further addition of ammonium sulphate to a final concentration of 75% (w/v) was carried out to precipitate EGFP from solution. The precipitate was resuspended in buffer A (5 ml) and the protein solution was then applied to a SP Superdex 200 gel filtration column (GE Healthcare) with elution monitored at 280 nm and 488 nm. The purified protein sample was finally stored in buffer A supplemented with 150 mM NaCl. A detailed description of absorption and fluorescence methods is provided in Methods S1.

### Protein crystallisation and structure determination

Purified EGFP (10 mg/ml in 50 mM Tris-HCl, pH 8.0 and 150 mM NaCl) was screened for crystal formation by the sitting drop vapour diffusion method with incubation at 4°C. Drops were set up with equal volumes of protein and precipitant solutions (0.5 µl each). Crystals of EGFP were obtained from 0.1 M MES/NaOH, pH 6.5, 200 mM calcium acetate and 20% (w/v) PEG 8000. A crystal was transferred to mother liquor supplemented with 13% (w/v) PEG 200 as a cryoprotectant and vitrified. Data were collected on beamline I02 at the Diamond Light Source, Harwell, UK. Usable diffraction was recorded up to a resolution of 1.35 Å. Data were reduced with the XIA2 package [21], space group assignment was done by POINTLESS [22], scaling and merging were completed with SCALA [22] and TRUNCATE [23]. Initial molecular replacement for the EGFP structure was performed using a previously determined GFP structure (PDB entry 2HQZ) as the search model, using MOLREP [24]. The structure for EGFP was adjusted manually using COOT [25] and refinement of the completed molecule was carried out using REFMAC [26]. Protein atoms were refined anisotropically, but residues shown as sphericity outliers by REFMAC were refined isotropically. All non-protein atoms were refined isotropically. The above routines were used as the CCP4 package [23] (www.ccp4.ac.uk). Graphical representations were made with PyMOL Molecular Graphics System, Schrödinger, LLC.

## Supporting Information

Figure S1 **Size exclusion chromatography of EGFP.** Samples of EGFP were applied to a Superdex™ 75 gel filtration column and the elution monitored at 488 nm. Protein concentrations of 10 µM (solid black line), 25 µM (long dashed line), 50 µM (medium dashed line) or 100 µM (short dashed line) were applied to the column. A small decrease in peak elution volume (∼0.15 ml) was observed with increasing protein concentration (from 10–100 µM), corresponding to a small increase in apparent molecular weight (∼24.6–∼26.8 kDa). The apparent molecular weight was still very close to the theoretical molecular weight calculated from the amino acid sequence (26941 Da). The elution peak was non-symmetrical, suggesting there was more than one oligomeric species present in dynamic equilibrium with the monomeric form; this is consistent with previous observations that wt GFP is largely monomeric with a weak tendency to dimerise.
(TIFF)

Figure S2 **Rationale behind modelling of E222 as a double conformer.** Modelling of residue E222 as either the single conformer A (A), the single conformer B (B) or as a double conformer as observed in PDB entry 2Y0G [S1] (C) does not fully satisfy the electron density difference map. Modelling of the double conformer as seen here (D) satisfies the electron density.
(TIFF)

Figure S3 **Structural comparison of E222 double conformers in the present structure (4EUL) and 2Y0G.** (A) Overlay of all four conformers from 4EUL and 2Y0G. Conformer A and B from 4EUL are coloured green and yellow, respectively. Conformer A and B from 2Y0G [S1] are coloured orange and blue, respectively. The significant difference in placement of the side chain atoms for E22 conformer B in 4EUL in comparison to conformer B in 2Y0G are clearly seen. For clarity, the double conformers of E222 for 2Y0G (B) and 4EUL (C) have also been shown.
(TIFF)

Figure S4 **Residues with multiple conformers in EGFP.** Electron density difference maps and residues in EGFP with multiple conformers are shown as sticks and coloured green, yellow or grey for conformer A, B or C respectively.
(TIFF)

Figure S5 **Structural comparison of L18 and L44 in the present structure (4EUL) and 2Y0G.** Overlay of the single observed conformer of Leu18 (A) or L44 (B) for 2Y0G [S1] with conformer A (green) and conformer B (red) observed in the present study (4EUL).
(TIFF)

Table S1 **Secondary structure assignment for EGFP and wt GFP.**
(DOCX)

Methods S1 **Supporting Methods.**
(DOCX)

References S1 **Supporting References.**
(DOCX)

## References

[1] Shimomura O, Johnson FH, Saiga Y (1962) Extraction, purification and properties of aequorin, a bioluminescent protein from the luminous hydromedusan, Aequorea. J Cell Comp Phys 59: 223–239. 10.1002/jcp.1030590302 13911999

[2] Chalfie M, Tu Y, Euskirchen G, Ward WW, Prasher DC (1994) Green fluorescent protein as a marker for gene expression. Science 263: 802–805. 8303295 10.1126/science.8303295

[3] Tsien RY (1998) The green fluorescent protein. Ann Rev Biochem 67: 509–544. 9759496 10.1146/annurev.biochem.67.1.509

[4] Zhang J, Campbell RE, Ting AY, Tsien RY (2002) Creating new fluorescent probes for cell biology. Nat Rev Mol Cell Biol 3: 906–918. 12461557 10.1038/nrm976

[5] Pakhomov AA, Martynov VI (2008) GFP family: structural insights into spectral tuning. Chem Biol 15: 755–764. 18721746 10.1016/j.chembiol.2008.07.009

[6] Zhang L, Patel HN, Lappe JW, Wachter RM (2006) Reaction progress of chromophore biogenesis in green fluorescent protein. J Am Chem Soc 128: 4766–4772. 16594713 10.1021/ja0580439

[7] Yang F, Moss LG, Phillips GN Jr (1996) The molecular structure of green fluorescent protein. Nat Biotech 14: 1246–1251. 10.1038/nbt1096-1246 9631087

[8] Ormo M, Cubitt AB, Kallio K, Gross LA, Tsien RY, et al (1996) Crystal structure of the Aequorea victoria green fluorescent protein. Science 273: 1392–1395. 8703075 10.1126/science.273.5280.1392

[9] Heim R, Prasher DC, Tsien RY (1994) Wavelength mutations and posttranslational autoxidation of green fluorescent protein. Proc Natl Acad Sci USA 91: 12501–12504. 7809066 10.1073/pnas.91.26.12501 PMC45466

[10] Lippincott-Schwartz J, Patterson GH (2003) Development and use of fluorescent protein markers in living cells. Science 300: 87–91. 12677058 10.1126/science.1082520

[11] Cormack BP, Valdivia RH, Falkow S (1996) FACS-optimized mutants of the green fluorescent protein (GFP). Gene 173: 33–38. 8707053 10.1016/0378-1119(95)00685-0

[12] Yang TT, Cheng L, Kain SR (1996) Optimized codon usage and chromophore mutations provide enhanced sensitivity with the green fluorescent protein. Nucleic Acids Res 24: 4592–4593. 8948654 10.1093/nar/24.22.4592 PMC146266

[13] Royant A, Noirclerc-Savoye M (2011) Stabilizing role of glutamic acid 222 in the structure of Enhanced Green Fluorescent Protein. J Struc Biol 174: 385–390. 10.1016/j.jsb.2011.02.004 21335090

[14] Patterson GH, Knobel SM, Sharif WD, Kain SR, Piston DW (1997) Use of the green fluorescent protein and its mutants in quantitative fluorescence microscopy. Biophys J 73: 2782–2790. 9370472 10.1016/S0006-3495(97)78307-3 PMC1181180

[15] Budisa N, Pal PP, Alefelder S, Birle P, Krywcun T, et al (2004) Probing the role of tryptophans in Aequorea victoria green fluorescent proteins with an expanded genetic code. Biol Chem 385: 191–202. 15101562 10.1515/BC.2004.038

[16] Steiner T, Hess P, Bae JH, Wiltschi B, Moroder L, et al (2008) Synthetic biology of proteins: tuning GFPs folding and stability with fluoroproline. PloS One 3: e1680. 18301757 10.1371/journal.pone.0001680 PMC2243022

[17] Heim R, Cubitt AB, Tsien RY (1995) Improved green fluorescence. Nature 373: 663–664. 10.1038/373663b0 7854443

[18] Brejc K, Sixma TK, Kitts PA, Kain SR, Tsien RY, et al (1997) Structural basis for dual excitation and photoisomerization of the Aequorea victoria green fluorescent protein. Proc Natl Acad Sci U S A 94: 2306–2311. 9122190 10.1073/pnas.94.6.2306 PMC20083

[19] Shu X, Kallio K, Shi X, Abbyad P, Kanchanawong P, et al (2007) Ultrafast excited-state dynamics in the green fluorescent protein variant S65T/H148D. 1. Mutagenesis and structural studies. Biochemistry 46: 12005–12013. 17918959 10.1021/bi7009037 PMC2536499

[20] Stepanenko OV, Verkhusha VV, Kazakov VI, Shavlovsky MM, Kuznetsova IM, et al (2004) Comparative studies on the structure and stability of fluorescent proteins EGFP, zFP506, mRFP1, “dimer2”, and DsRed1. Biochemistry 43: 14913–14923. 15554698 10.1021/bi048725t

[21] Winter G (2010) xia2: an expert system for macromolecular crystallography data reduction. J App Crystallogr 43: 186–190.

[22] Evans PR (2006) Scaling and assessment of data quality. Acta Cryst D62: 72–82. 10.1107/S0907444905036693 16369096

[23] CCP4 (1994) The CCP4 suite: programs for protein crystallography. Acta Crystallogr D Biol Crystallogr 50: 760–763. 15299374 10.1107/S0907444994003112

[24] Lebedev AA, Vagin AA, Murshudov GN (2008) Model preparation in MOLREP and examples of model improvement using X-ray data. Acta Crystallogr D Biol Crystallogr 64: 33–39. 18094465 10.1107/S0907444907049839 PMC2394799

[25] Emsley P, Cowtan K (2004) Coot: model-building tools for molecular graphics. Acta Crystallogr D Biol Crystallogr 60: 2126–2132. 15572765 10.1107/S0907444904019158

[26] Murshudov GN, Vagin AA, Dodson EJ (1997) Refinement of macromolecular structures by the maximum-likelihood method. Acta Crystallogr D Biol Crystallogr 53: 240–255. 15299926 10.1107/S0907444996012255

