# Supplementary figures and images for: Crystal Structure of Enhanced Green Fluorescent Protein to 1.35 Å Resolution Reveals Alternative Conformations for Glu222
Source: PLoS One. 2012 Oct 16;7(10):e47132. doi: 10.1371/journal.pone.0047132 (PMC3473056; doi:10.1371/journal.pone.0047132)

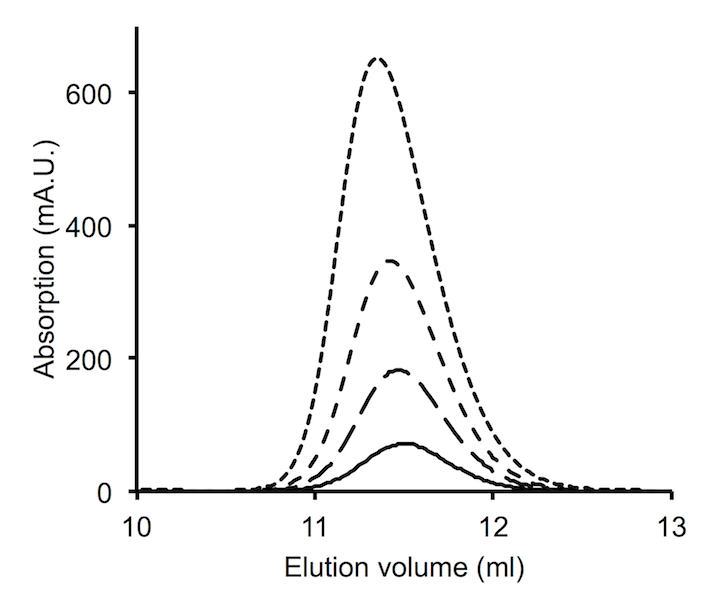

Supplement: Figure S1 — Size exclusion chromatography of EGFP. Samples of EGFP were applied to a Superdex™ 75 gel filtration column and the elution monitored at 488 nm. Protein concentrations of 10 µM (solid black line), 25 µM (long dashed line), 50 µM (medium dashed line) or 100 µM (short dashed line) were applied to the column. A small decrease in peak elution volume (∼0.15 ml) was observed with increasing protein concentration (from 10–100 µM), corresponding to a small increase in apparent molecular weight (∼24.6–∼26.8 kDa). The apparent molecular weight was still very close to the theoretical molecular weight calculated from the amino acid sequence (26941 Da). The elution peak was non-symmetrical, suggesting there was more than one oligomeric species present in dynamic equilibrium with the monomeric form; this is consistent with previous observations that wt GFP is largely monomeric with a weak tendency to dimerise. (TIFF) [file pone.0047132.s001.tiff]

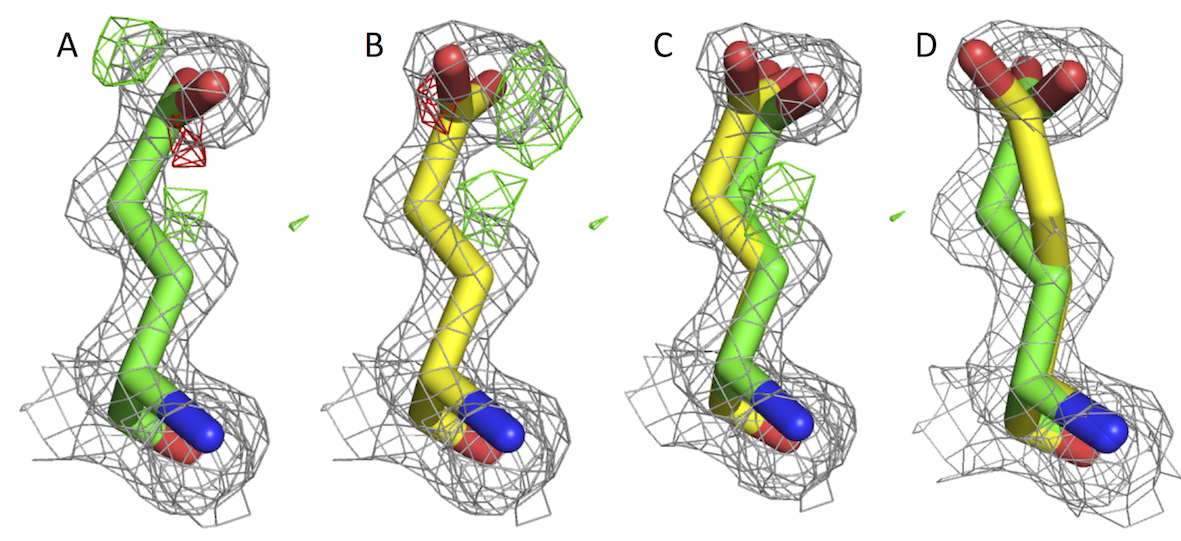

Supplement: Figure S2 — Rationale behind modelling of E222 as a double conformer. Modelling of residue E222 as either the single conformer A (A), the single conformer B (B) or as a double conformer as observed in PDB entry 2Y0G [S1] (C) does not fully satisfy the electron density difference map. Modelling of the double conformer as seen here (D) satisfies the electron density. (TIFF) [file pone.0047132.s002.tiff]

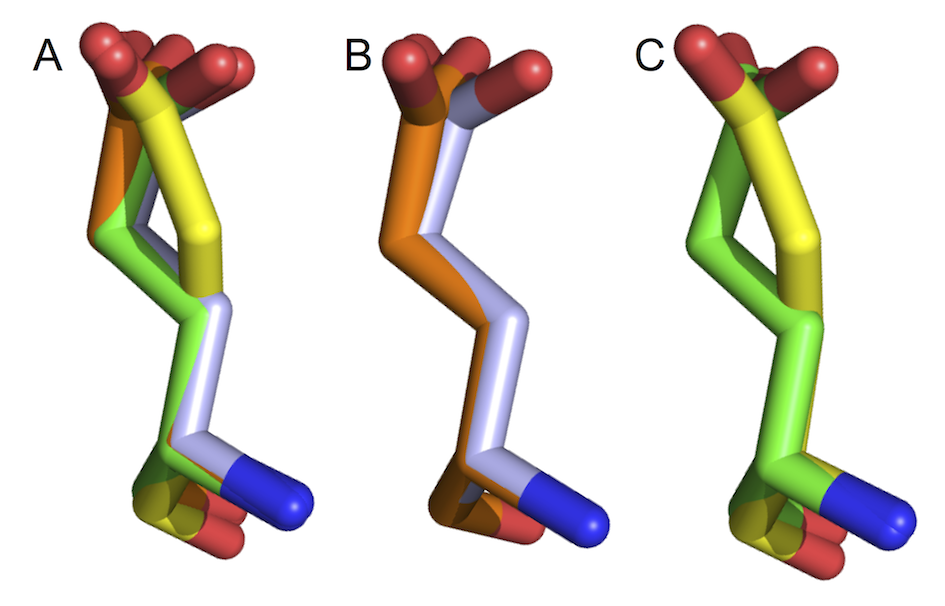

Supplement: Figure S3 — Structural comparison of E222 double conformers in the present structure (4EUL) and 2Y0G. (A) Overlay of all four conformers from 4EUL and 2Y0G. Conformer A and B from 4EUL are coloured green and yellow, respectively. Conformer A and B from 2Y0G [S1] are coloured orange and blue, respectively. The significant difference in placement of the side chain atoms for E22 conformer B in 4EUL in comparison to conformer B in 2Y0G are clearly seen. For clarity, the double conformers of E222 for 2Y0G (B) and 4EUL (C) have also been shown. (TIFF) [file pone.0047132.s003.tiff]

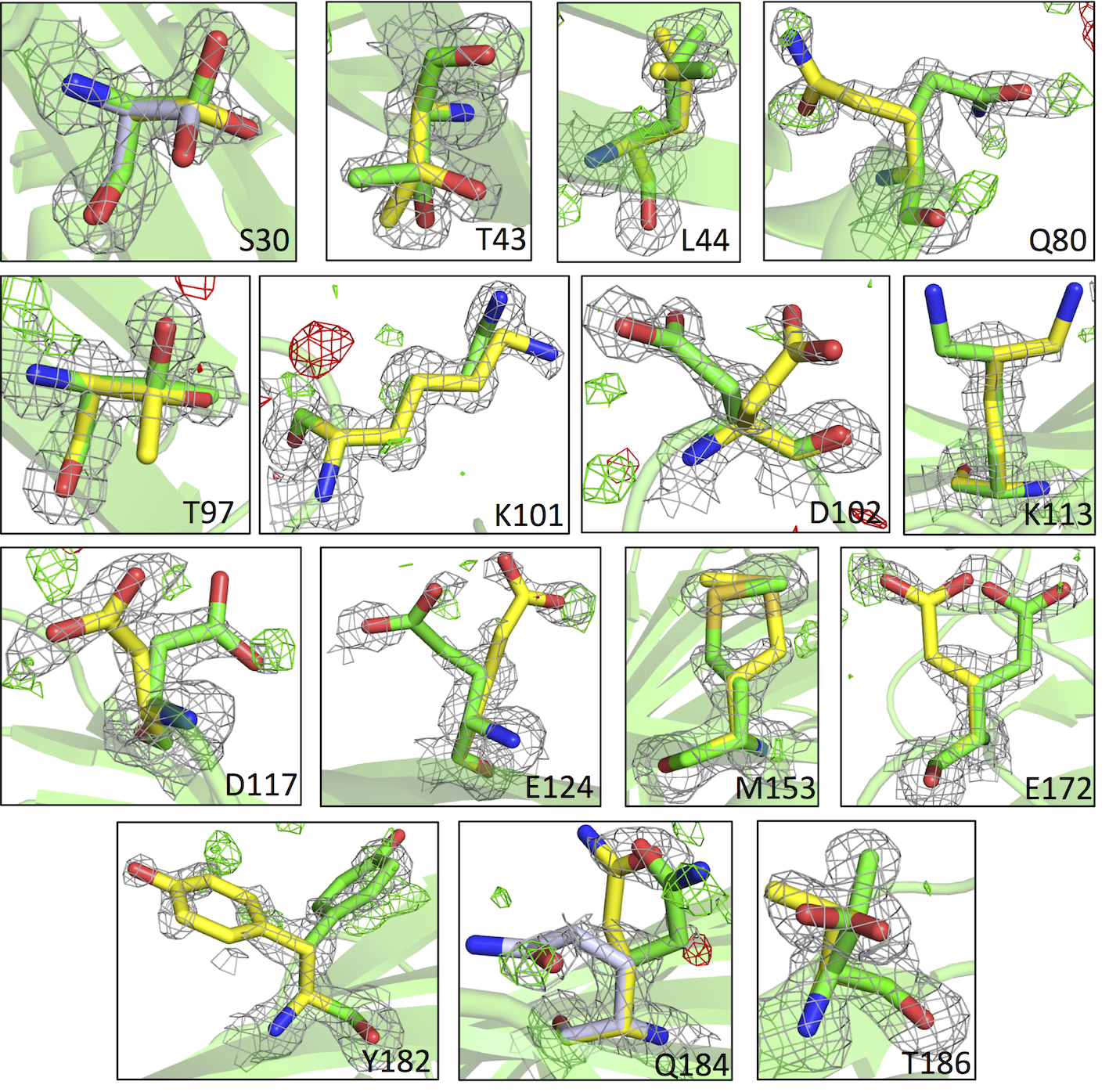

Supplement: Figure S4 — Residues with multiple conformers in EGFP. Electron density difference maps and residues in EGFP with multiple conformers are shown as sticks and coloured green, yellow or grey for conformer A, B or C respectively. (TIFF) [file pone.0047132.s004.tiff]

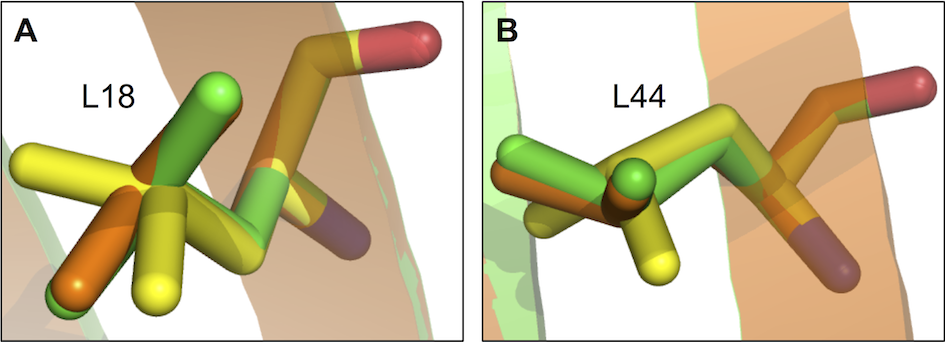

Supplement: Figure S5 — Structural comparison of L18 and L44 in the present structure (4EUL) and 2Y0G. Overlay of the single observed conformer of Leu18 (A) or L44 (B) for 2Y0G [S1] with conformer A (green) and conformer B (red) observed in the present study (4EUL). (TIFF) [file pone.0047132.s005.tiff]
