# Supplementary material for: Crystal Structure of Enhanced Green Fluorescent Protein to 1.35 Å Resolution Reveals Alternative Conformations for Glu222
Source: PLoS One. 2012 Oct 16;7(10):e47132. doi: 10.1371/journal.pone.0047132 (PMC3473056; doi:10.1371/journal.pone.0047132)
Supplement: Table S1 — Secondary structure assignment for EGFP and wt GFP. (DOCX) [file pone.0047132.s006.docx]

**Supporting Table 1. Secondary structure assignment for EGFP and wt GFP.**

| EGFP | | wt GFP^a^ | |
| --- | --- | --- | --- |
| Residue Range^b^ | Secondary structure^c^ | Residue Range^b^ | Secondary Structure^c^ |
| E5 - F8 | 3_10_ helix | E5 - F8 | 3_10_ helix |
| V12 -V22 | β strand | V12 - V22 | β strand |
| H25 - D36 | β strand | H25 - D36 | β strand |
| A37 - Y39 | 3_10_ helix | - | - |
| K41 - C48 | β strand | K41 - C48 | β strand |
| P56 - L60 | 3_10_ helix | P56 - L60 | 3_10_ helix |
| Q69 - F71 | 3_10_ helix | Q69 - F71 | 3_10_ helix |
| D76 - H81 | 3_10_ helix | D76 - H81 | 3_10_ helix |
| F83 - F86 | α helix | F83 - S86 | α helix |
| Y92 - F100 | β strand | Y92 - F100 | β strand |
| N105-E115 | β strand | N105-E115 | β strand |
| T118 - I128 | β strand | T118 - I128 | β strand |
| N149 - D155 | β strand | H148 - D155 | β strand |
| K156 - K158 | 3_10_ helix | K156 - K158 | 3_10_ helix |
| G160 - E170 | β strand | G160 - E170 | β strand |
| V176 - P187 | β strand | V176 - P187 | β strand |
| H199 - S208 | β strand | H199 - S208 | β strand |
| H217 - A227 | β strand | H217 - A227 | β strand |

^a^ wt GFP PDB: 1GFL [[2](#_ENREF_2)]
^b^ Residue numbering as for wt GFP [[2](#_ENREF_2)]
^c^ Secondary structure assignment performed by DSSP
