# Supplementary material for: Crystal Structure of Enhanced Green Fluorescent Protein to 1.35 Å Resolution Reveals Alternative Conformations for Glu222
Source: PLoS One. 2012 Oct 16;7(10):e47132. doi: 10.1371/journal.pone.0047132 (PMC3473056; doi:10.1371/journal.pone.0047132)
Supplement: Methods S1 — Supporting Methods. (DOCX) [file pone.0047132.s007.docx]

**Supporting Methods**

*Absorbance spectroscopy:* UV-visible absorbance spectra were recorded with a Hewlett Packard diode array spectrophotometer with a 1 cm path length. Purified protein was diluted into 50 mM Tris-HCl pH 8.0 at 25°C supplemented with 150 mM NaCl.

*Quantum yield determination:* The quantum yield for EGFP was calculated using fluorescein as a reference. Fluorescein (in 0.1 M NaOH) and EGFP (in 50 mM Tris-HCl pH 8.0 at 25°C) were prepared with A_488_ of 0.02 in a 10 mm pathlength cuvette. Emission spectra were measured after excitation at 488 nm using a 5 x 5 mm pathlength cuvette with an excitation and emission band pass of 2.5 nm. Integrated emission intensity between 500 and 650 nm was calculated and used in the following formula to generate quantum yield values

φ_x_ = φ_st_ . (Area_x_/Area_st_).(η^2^_x_/η^2^_st_)

where the φ_x_ and φ_st_ refer to the fluorescence quantum yield of the sample and fluorescein standard, respectively. Area_x_ and Area_st_ are the integrated emission intensities for the sample and fluorescein standard, respectively. η_x_ and η_st_ is the refractive index of the solvent for the sample and fluorescein standard, respectively. The refractive index correction here was negligible as 0.1 M NaOH and aqueous buffers differ in refractive index by <1%.

*Fluorescence lifetime determination:* Fluorescence lifetimes were determined by a time correlated single photon counting (TCSPC) technique using an FLS-920 fluorometer (Edinbugh Instruments) with a hydrogen nF900 nanosecond flashlamp excitation source, under 0.4 bar pressure, and a PMT-Hamamatsu R2658P detector. Samples were measured across 512 channels (0.039 ns/channel) until one channel reached a maximum photon count of 500 after excitation at 488 nm and emission monitored at 511 nm. An instrument response function (IRF) was measured with every sample measurement for reconvolution of the data for exponential curve fitting. Protein samples were diluted into 50 mM Tris-HCl pH 8.0 to a final concentration of 6 μM in a 1 cm pathlength cuvette for measurement. The fluorescence lifetime data were fit to a single exponential decay after IRF reconvolution of the data using Edinburgh instruments F900 software.

*Size exclusion chromatography:* Gel filtration standards (Biorad) were applied to a Superdex™ 200 column (20 ml bed volume, 0.5 ml/min flow rate), as per the manufacturers guidelines, with protein elution monitored at 280 nm. A standard curve was generated from the plot of LogMw against K_av_, where K_av_ = (V_e_-V_o_)/(V_t_-V_o_), V_e_ is the elution volume, V_o_ is the void volume and V_t_ is the total volume. Protein samples were prepared in 50 mM Tris-HCl, pH 8.0, 150 mM NaCl in final concentrations of 25, 50 or 100 μM and applied to the Superdex™ 200 column equilibrated with the protein preparation buffer, with protein elution monitored by absorbance at 488 nm. Elution volumes (V_e_) were determined for each sample and K_av_ values calculated. Using the standard curve estimated molecular weights could be determined for each protein sample.
