# Supplementary material for: Crystal Structure of Enhanced Green Fluorescent Protein to 1.35 Å Resolution Reveals Alternative Conformations for Glu222
Source: PLoS One. 2012 Oct 16;7(10):e47132. doi: 10.1371/journal.pone.0047132 (PMC3473056; doi:10.1371/journal.pone.0047132)
Supplement: References S1 — Supporting References. (DOCX) [file pone.0047132.s008.docx]

**Supporting References**

1. Royant A, Noirclerc-Savoye M (2011) Stabilizing role of glutamic acid 222 in the structure of Enhanced Green Fluorescent Protein. J Struc Biol 174: 385-390.

2. Yang F, Moss LG, Phillips GN, Jr. (1996) The molecular structure of green fluorescent protein. Nature Biotech 14: 1246-1251.
